# SFPQ promotes an oncogenic transcriptomic state in melanoma

**DOI:** 10.1038/s41388-021-01912-4

**Published:** 2021-07-03

**Authors:** O. Bi, C. A. Anene, J. Nsengimana, M. Shelton, W. Roberts, J. Newton-Bishop, J. R. Boyne

**Affiliations:** 1 School of Applied Sciences, University of Huddersfield, Huddersfield, UK; 2 Centre for Cancer Genomics and Computational Biology, Barts Cancer Institute, Queen Mary University of London, London, UK; 3 Population Health Sciences Institute, Faculty of Medical Sciences, Newcastle University, Newcastle, UK; 4 School of Clinical and Applied Science, Leeds Beckett University, Leeds, UK; 5 University of Leeds School of Medicine, Leeds, UK

**Keywords:** Skin cancer, Clinical genetics

## Abstract

The multifunctional protein, splicing factor, proline- and glutamine-rich (SFPQ) has been implicated in numerous cancers often due to interaction with coding and non-coding RNAs, however, its role in melanoma remains unclear. We report that knockdown of SFPQ expression in melanoma cells decelerates several cancer-associated cell phenotypes, including cell growth, migration, epithelial to mesenchymal transition, apoptosis, and glycolysis. RIP-seq analysis revealed that the SFPQ-RNA interactome is reprogrammed in melanoma cells and specifically enriched with key melanoma-associated coding and long non-coding transcripts, including *SOX10, AMIGO2* and *LINC00511* and in most cases SFPQ is required for the efficient expression of these genes. Functional analysis of two SFPQ-enriched lncRNA, *LINC00511* and *LINC01234*, demonstrated that these genes independently contribute to the melanoma phenotype and a more detailed analysis of *LINC00511* indicated that this occurs in part via modulation of the miR-625-5p/*PKM2* axis. Importantly, analysis of a large clinical cohort revealed that elevated expression of SFPQ in primary melanoma tumours may have utility as a prognostic biomarker. Together, these data suggest that SFPQ is an important driver of melanoma, likely due to SFPQ–RNA interactions promoting the expression of numerous oncogenic transcripts.

## Introduction

At the molecular level, melanoma comprises a heterogeneous group of disorders that harbour distinct aberrations in diverse cellular processes [1–3]. Such heterogeneity suggests that multiple mechanisms are involved in disease aetiology, and this is reflected in the contribution of both different mutations and differential gene and protein expression associated with melanoma transformation and progression [4].

Splicing factor proline- and glutamine-rich (SFPQ) is a multifunctional DNA- and RNA-binding protein that regulates numerous cellular mechanisms, including RNA-processing, transcription, DNA damage response and innate immunity [5–9]. Aberrant SFPQ function is associated with the aetiology of neurodegenerative disorders [10–12] and colorectal [13], hepatocellular [14], renal [15], Chronic Myeloid Leukaemia [16] and prostate cancer [17]. Intriguingly, it appears that SFPQ-long non-coding RNA (lncRNA) interactions are responsible for pathological changes in numerous cancers [14, 15, 18] including breast [19, 20] and ovarian tumours [21]. Despite comprising a family of up to 90,000 transcripts [22], the majority of lncRNAs are incompletely annotated and remain poorly understood at the functional level. However, dysregulated expression of lncRNA has been shown to correlate with poor outcome across a range on neoplasms, with increasing numbers of lncRNA being implicated in driving metastasis [23–25].

Here we investigated the role of SFPQ in melanoma cells and showed that expression of SFPQ contributes to melanoma cell growth, migration, apoptosis, glycolysis, and epithelial to mesenchymal transition (EMT). Investigation and interrogation of the SFPQ-RNA interactome in primary melanocytes compared with melanoma cells via RNA-immunoprecipitations (RIP)-seq revealed a dramatic reprogramming in melanoma that favoured coding and non-coding transcripts associated with the promotion of metastatic disease. Furthermore, knockdown of SFPQ led to a significant decrease in many of these enriched transcripts, including the lncRNA, *LINC00511*, which we showed contributes to the melanoma cell phenotype. Finally, analysis of our melanoma patient data revealed that high levels of SFPQ expression in tumours negatively correlate with patient survival, suggesting that SFPQ may have utility as a prognostic biomarker.

## Results

### SFPQ knockdown decelerates cancer cell phenotype in melanoma

**Fig. 1:**
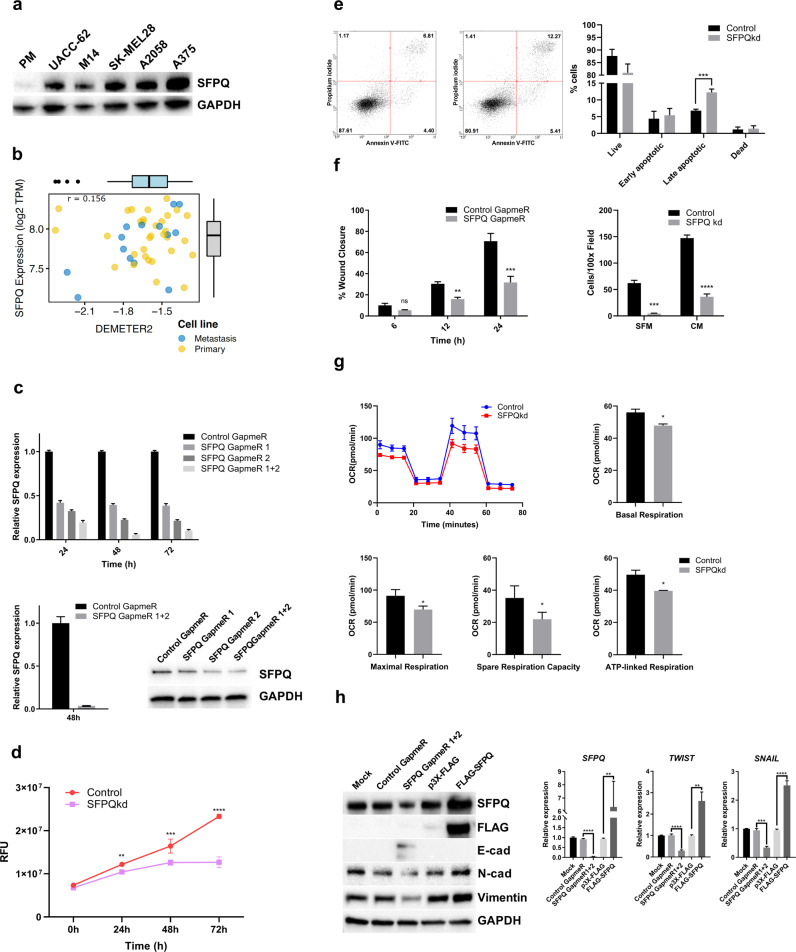
SFPQ expression promotes the cancer phenotype in melanoma. **a** Total protein was isolated from primary melanocytes (PM) and melanoma cell lines prior to analysis of SFPQ expression via immunoblot, *n* = 3. **b** SFPQ dependency score (*x*-axis) was compared with SFPQ expression levels (*y*-axis) in melanoma cell lines. Within the plot, lower DEMETER2 scores correspond to more dependency on SFPQ, and positive values indicate no dependence. **c** Total RNA was extracted from A2058 cells transiently transfected with SFPQ-specific GapmeR or control for 24, 48 and 72 h prior to generation of cDNA and analysis of SFPQ expression via qPCR, *n* = 3. **d** Viable growth of A2058 cells transiently transfected with the indicated GapmeR and cultured for 24, 48 and 72 h was determined using CellTitre-Glo^®^, *n* = 3. **e** A2058 cells were transiently transfected with SFPQ-specific GapmeR or control and cultured for 48 h prior to staining with Annexin V-FITC and Propridium iodide and analysis via FACS, *n* = 3. **f** A2058 cells were transfected with SFPQ-specific GapmeR or control and cultured for 48 h prior to analysis of cell migration via wound healing (left panel) and transwell (right panel) assay, *n* = 3. **g** A2058 cells were transfected with SFPQ-specific GapmeR or control and cultured for 48 h prior to analysis of cellular respiration using the Cell Mito Stress Test kit, *n* = 3. **h** A2058 cells were transiently transfected with SFPQ-specific GapmeRs, pFLAG-SFPQ, or corresponding scramble GapmeR and parent vector controls and cultured for 48 h, prior to isolation of total protein and RNA and analysis of indicated EMT markers via western blot and qRT-PCR, respectively, *n* = 3.

To investigate the role of SFPQ in melanoma, we analysed SFPQ protein expression in untransformed primary human melanocytes (PM) alongside a range of melanoma cell lines. As can be seen in Fig. 1a, we observed a significant increase in SFPQ expression in each melanoma cell line compared with PM. SFPQ has been identified as an essential melanoma gene in a CRISPR genome-wide loss of function screen [26], and analysis of this dataset revealed that melanoma cell lines (*n* = 46) show high dependency (DEMETER2 scores) on SFPQ regardless of its expression (i.e., poor correlation between dependency score and expression levels) (Fig. 1b). Together, these data suggest that SFPQ may actively drive melanoma development. To test this, we explored if SFPQ expression was required for ‘cancer cell phenotypes’, in vitro, including increased cell growth, cell migration, EMT and decreased apoptosis and oxidative phosphorylation (OXPHOS). To this end, SFPQ expression was knocked down using SFPQ-specific GapmeR antisense oligonucleotides (ASOs) and decreased levels of SFPQ mRNA and protein confirmed via qRT-PCR and western blot, respectively (Fig. 1c). The impact of SFPQ knockdown on viable cell growth (Fig. 1d), cell death (Fig. 1e) and cell migration (Fig. 1f) was then determined and in each case, we observed a significant shift away from a cancer cell phenotype. Given the importance of dysregulated respiration in melanoma biology we also investigated if SFPQ knockdown affected cellular respiration via real-time analysis of oxygen consumption rate (OCR). Data in Fig. 1g show that knockdown of SFPQ led to a significant decrease in basal respiration, maximal respiration, ATP production and spare respiration capacity, suggesting impaired OXPHOS in cells with decreased expression of SFPQ. Finally, we investigated the impact of SFPQ knockdown and overexpression on several EMT markers and observed a decrease in the expression of mesenchymal markers following knockdown of SFPQ and an increase in cells where SFPQ expression was increased (Fig. 1h). Together, these data demonstrate that knockdown of SFPQ in melanoma cells results in a wide-ranging attenuation of the cancer cell phenotype.

### Comparative analysis of SFPQ-RNA interactome in primary melanocytes and melanoma cells

**Fig. 2:**
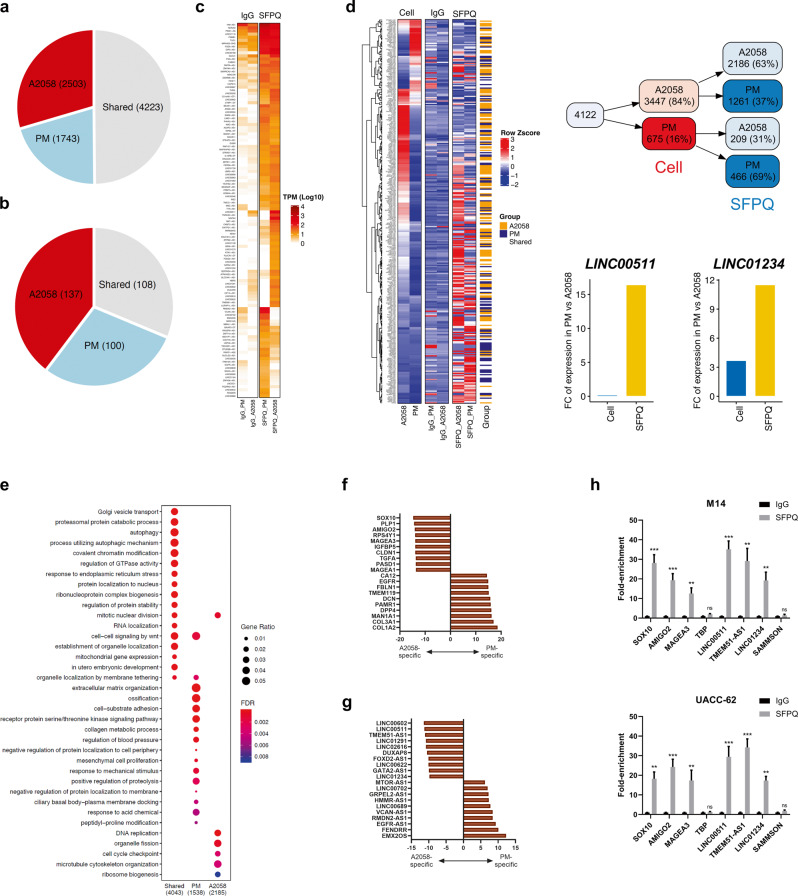
SFPQ–RNA interactome is reprogrammed in A2058 melanoma cells. **a** Pie chart depicts the proportion of all interaction RNA transcripts shared between PM and A2058, specific to PM or specific to A2058. **b** Pie chart depicts the proportion of non-coding interacting RNA transcripts shared between PM and A2058, specific to PM or specific to A2058. **c** Heatmap of expression profile of all identified lncRNA interactors across IgG control and SFPQ-IP. Within the heatmap, orange colour indicates enrichment in SFPQ-IP compared to IgG and white colour indicates no enrichment. **d** Heat map of relative expression profile of identified lncRNA interactors across whole-cell RNA (A2058 and PM), IgG control and SFPQ-IP. Within the heatmap, red colour indicates high expression relative to whole-cell RNA and blue colour indicates low expression (left panel). Distribution plots SFPQ-specific interactions against cell-type expression levels. Within the plot, the left panel (white box) is the total number of SFPQ interactors, middle panel (Cell) indicates the proportion of SFPQ interactors in terms of cell-specific expression. The right panel (SFPQ) shows the distribution of SFPQ-specific interactions by cell type (upper right panel). A2058 and PM data were analysed to determine the fold-increase in relative cell expression compared with fold-increase in relative SFPQ-enrichment for the A2058-specific SFPQ-enriched lncRNAs, *LINC00511* and *LINC01234* (lower right panel). **e** Cluster comparison plot of enriched biological processes for transcripts shared between PM and A2058, specific to PM or specific to A2058. Gene ratio genes in ontology/total gene set and FDR false discovery rate. **f** SFPQ-enriched mRNA transcripts were ranked based on the specificity of enrichment in either PM or A2058 melanoma cells. **g** SFPQ-enriched lncRNA transcripts were ranked based on the specificity of enrichment in either PM or A2058 melanoma cells. **h** SFPQ-RNA interactions identified in A2058 via RIP-seq were confirmed via RNA-IP and qRT-PCR in M14 and UACC-62 melanoma cells, *n* = 3.

Given data demonstrating a role for SFPQ in melanoma cell phenotype, we were keen to characterise the SFPQ-RNA interactome in PM and establish if this was reprogrammed in melanoma cells. To this end, PM and A2058 melanoma cells were lysed, and RIP performed using either an SFPQ-specific antibody or IgG-antibody isotype control (Fig. S1A), followed by analysis via RNA-seq. Initially, we identified all transcripts that were (i) significantly enriched with SFPQ in PM compared with A2058 cells; (ii) enriched with SFPQ in both PM and A2058 cells; (iii) significantly enriched with SFPQ in A2058 cells compared with PM (Fig. 2a). Most SFPQ-enriched transcripts comprised mRNAs, with 8124 unique peaks called, however, further analysis revealed 345 SFPQ–lncRNA interactions across both cell backgrounds (Fig. 2b). We observed significant differential enrichment of both mRNA and lncRNA transcripts between PM and A2058 cells and while the RNA biotype with shared enrichment included significantly more genes, the non-coding RNA (ncRNA) biotype was more significant in A2058, suggesting specific reprogramming of SFPQ–lncRNA interactions in melanoma. (Fig. 2c). To determine if baseline expression of these differentially enriched genes in PM and A2058 transcriptomes was influencing SFPQ interaction, whole-cell RNA expression profiles were integrated with RIP-Seq data with unsupervised clustering. We observed no association between whole-cell RNA transcript abundance and the specificity of the SFPQ–RNA interactions (Fig. 2d and Fig. S1B). Gene ontology (GO) analysis was then performed to identify enriched biological processes for PM-specific and A2058-specific SFPQ–mRNA interactors, respectively. Interestingly, PM-specific targets were significantly enriched for GO terms broadly associated with extracellular matrix (ECM) organisation, whereas A2058-specific targets were significantly associated with GO terms relating to the positive regulation of the cell cycle (Fig. 2e).

Given the widely documented relationship between SFPQ and lncRNAs in several cancers, including melanoma, SFPQ interactors were also ranked according to their PM- and A2058-specificity (Fig. 2f, g). Discerning the functional importance of enriched lncRNA gene lists from RNA-seq studies such as the one reported here is problematic, as the vast majority of lncRNAs remain functionally uncharacterised. However, it is interesting to note that the most PM-specific SFPQ-lncRNA interactors included genes associated with tumour suppressor function, such as *EMX2OS* and *FENDRR* [27, 28], whereas the most A2058-specific SFPQ-lncRNA interactors comprise genes widely reported as oncogenic, such as *LINC00511* [29–31]. We next sought to validate the top hits from our A2058 RIP-seq data in additional melanoma cell lines. To this end, SFPQ RIPs were performed using cell lysate isolated from the M14 and UACC-62 melanoma cell lines and enrichment with SFPQ assessed via qRT-PCR. As can be seen in Fig. 2h, the top mRNA and lncRNA targets enriched with SFPQ in the A2058 dataset were also significantly enriched with SFPQ in M14 and UACC-62 cells, whereas transcripts that were not present in our RIP-seq dataset did not exhibit significant enrichment with SFPQ. Collectively, these data show that compared with PM, SFPQ-RNA interactions in melanoma cells are reprogrammed and skewed towards oncogenic coding and non-coding transcripts.

### SFPQ-enriched melanoma-specific lncRNA contribute to the cancer phenotype

**Fig. 3:**
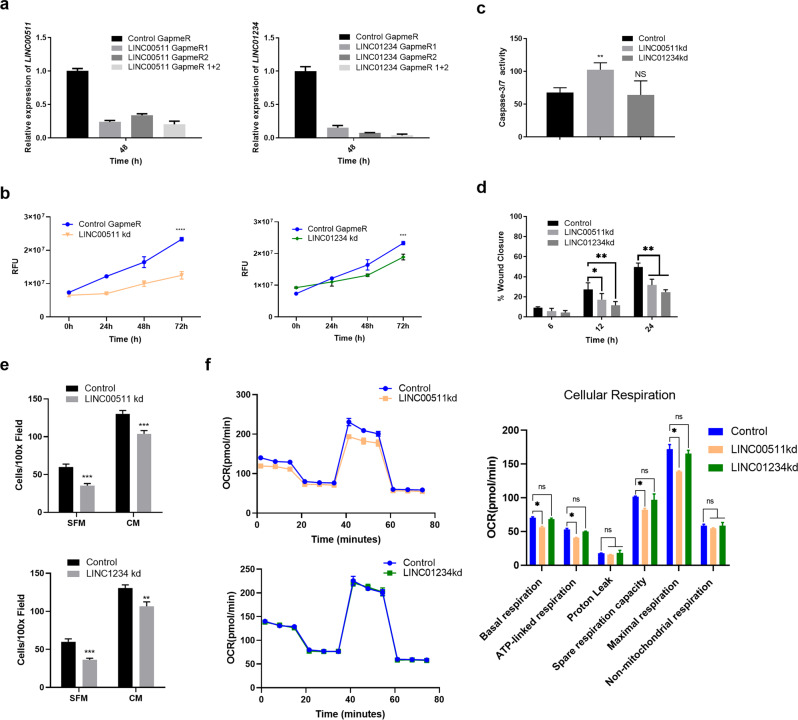
*LINC00511* and *LINC01234* contribute to the cancer phenotype in A2058 melanoma cells. **a** Total RNA was extracted from A2058 cells transiently transfected with the indicated GapmeR for 48 h prior to generation of cDNA and analysis of target gene expression via qPCR, *n* = 3. **b** Viable growth of A2058 cells transiently transfected with the indicated GapmeR and cultured for 24, 48 and 72 h was determined using CellTitre-Glo^®^, *n* = 3. **c** A2058 cells were transiently transfected with the indicated GapmeR and cultured for 48 h prior to addition of the Caspase-Glo® 3/7 reagent. Plates were incubated at room temperature for 30 min and luminescence recorded, *n* = 3. **d** A2058 cells were transfected with the indicated GapmeR and cultured for 48 h prior to analysis of cell migration via wound healing assay, *n* = 3. **e** A2058 cells were transfected with the indicated GapmeR and cultured for 48 h prior to analysis of cell migration via transwell assay, *n* = 3 **f** A2058 cells were transfected with the indicated GapmeR and cultured for 48 h prior to analysis of cellular respiration using the Cell Mito Stress Test kit, *n* = 3.

RIP-seq analysis identified 137 lncRNA that is enriched with SFPQ in A2058 melanoma cells. To establish if any novel melanoma-specific SFPQ-enriched lncRNA were functionally important for the cancer phenotype, we opted to investigate two SFPQ-enriched genes, *LINC00511* and *LINC01234*, which have recently been implicated in the progression of cancer, but to date have not been associated with melanoma. Initially, A2058 cells were transfected with either control, *LINC00511*- or *LINC01234-*specific GapmeR ASOs and knockdown of target transcript levels confirmed via qRT-PCR (Fig. 3a). We observed a marked reduction in *LINC00511-*depleted viable cell growth over 72 h and a lesser, but still significant reduction in growth kinetics following knockdown of *LINC01234* (Fig. 3b). Interestingly, caspase 3/7 activity was significantly increased following knockdown of *LINC00511*, but not in *LINC01234-*depleted cells (Fig. 3c). In contrast, we observed a significant decrease in cell migration following knockdown of either *LINC00511* or *LINC01234* (Figs. 3d, e). Finally, whereas knockdown of *LINC01234* had little impact on cellular respiration, *LINC00511*-depleted A2058 cells exhibited nominal but significant decreases for basal and maximal respiration, ATP-linked respiration, and spare respiration capacity (Fig. 3f).

### *LINC00511*-miR-625-5p-*PKM2* axis promotes glycolysis in melanoma

**Fig. 4:**
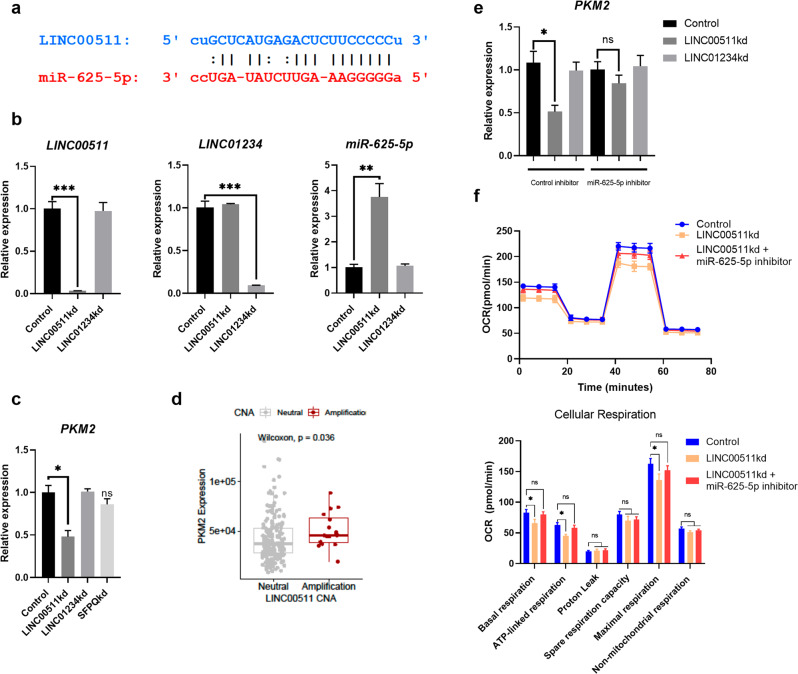
*LINC00511/miR-625-5p/PKM2* axis regulates cellular respiration in A2058 melanoma cells. **a** Analysis of HITS-CLIP-seq data identified miR-625-5p as a LINC00511 interaction partner. **b** Total RNA was extracted from A2058 cells transiently transfected with the indicated GapmeR for 48 h prior to generation of cDNA and analysis of target gene expression via qPCR (LINC00511 and LINC01234) and miRCURY LNA miRNA PCR (miR-625-5p), *n* = 3. **c** Total RNA was extracted from A2058 cells transiently transfected with the indicated GapmeR for 48 h prior to generation of cDNA and analysis of PKM2 expression via qPCR, *n* = 3. **d** TCGA-melanoma datasets were analysed to determine the impact of *LINC00511* CNV amplification on *PKM2* expression **e** Total RNA was extracted from A2058 cells transiently co-transfected with the indicated GapmeR alongside either miR-625-5p-specific miRCURY LNA power inhibitor or control for 48 h prior to generation of cDNA and analysis of *PKM2* expression via qPCR, *n* = 3. **f** A2058 cells were transiently co-transfected with the indicated GapmeR alongside either miR-625-5p-specific miRCURY LNA power inhibitor or control for 48 h prior to analysis of cellular respiration using the Cell Mito Stress Test kit, *n* = 3.

Given the pronounced effect of *LINC00511* knockdown in A2058 cells on cancer cell phenotype, we decided to investigate this gene further. To this end, publicly available Ago2-CLIP data [32] was analysed to identify miRNAs that interact with *LINC00511* and miR-625-5p identified as the top interactor (Fig. 4a), an observation supported by several recent articles demonstrating that *LINC00511* directly sponges miR-625-5p in renal, gastric and lung cancer [33–35]. To investigate if *LINC00511* is functioning to sponge miR-625-5p in melanoma, *LINC00511* was knocked down in A2058 cells, as described above, and miR-625-5p expression analysed via miRCURY LNA miRNA PCR Assay. As can be seen in Fig. 4b, depletion of *LINC00511* led to a significant increase in miR-625-5p transcript level, compared with control and knockdown of *LINC01234*, suggesting that *LINC00511* may sponge miR-625-5p in melanoma cells. Recently, miR-625-5p was reported to target the *PKM2* isoform of pyruvate kinase in melanoma to attenuate glucose consumption. To explore if *LINC00511* knockdown led to a decrease in the expression of *PKM2*, *LINC00511*-depleted A2058 melanoma cells were analysed via qRT-PCR and a significant decrease in *PKM2* expression was observed, whereas no decrease in *PKM2* expression was seen in either SFPQ- or *LINC01234*-depleted cells (Fig. 4c and Fig. S2). Consistently, analysis of the TCGA-melanoma dataset demonstrated that amplification of *LINC00511* significantly increased the expression of *PKM2* (Fig.4d). To investigate if the decrease in *PKM2* transcripts was due to impaired *LINC00511*-mediated sponging of miR-625-5p, *LINC00511*-depleted cells were co-transfected with miR-625-5p-specific inhibitor or scramble control and *PKM2* expression assessed, as described above. As can be seen in Fig. 4e, transfection with miR-625-5p inhibitor restored *PKM2* transcript abundance following *LINC00511* knockdown. Next, we utilised Seahorse XF analysis to determine if inhibition of miR-625-5p rescued the decrease in OCR in *LINC00511-*depleted cells. Data in Fig. 4f show that co-transfection of *LINC00511* and miR-625-5p inhibitor led to a partial but significant rescue of the diminished basal and maximal OCR observed following *LINC00511* knockdown. Together, these data demonstrate that expression of *LINC00511* in melanoma regulates the miR-625-5p/*PKM2* axis, which may explain *LINC00511*-dependent changes to OCR in A2058 melanoma cells.

### SFPQ regulates the expression of RNA interactors

**Fig. 5:**
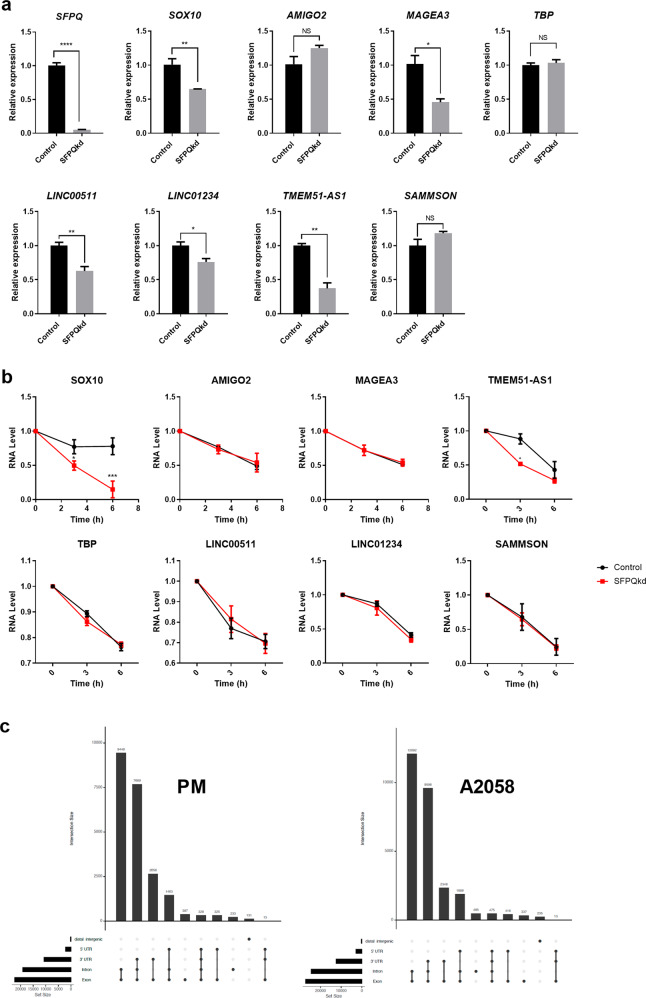
SFPQ enhances the expression of target RNAs. **a** Total RNA was extracted from A2058 cells transiently transfected with SFPQ-specific GapmeR or control for 48 h prior to generation of cDNA and analysis of target gene expression via qPCR, *n* = 3. **b** A2058 cells were transiently transfected with SFPQ-specific GapmeR or control for 48 h prior to the addition of actinomycin D (1 μg/ml). Total RNA was isolated from treated cells at 3 and 6 h, cDNA generated and used to analyse target genes via qPCR, *n* = 3. **c** ChIPseeker R package was used to annotate SFPQ enrichment on exon, intron, UTR, intergenic and promoter regions and data processed via the Integrative Genomics Viewer (IGV) to visualise the coverage of the reads across the identified genes.

To determine if SFPQ regulates the transcript levels of interacting RNAs in melanoma, SFPQ expression was knocked down in A2058 cells and expression of several SFPQ-enriched mRNA and lncRNA genes determined via qRT-PCR. As can be seen in Fig. 5a, knockdown of SFPQ led to a significant decrease in the transcript abundance of several, but not all, SFPQ-enriched RNAs. Specifically, we observed approximately two-fold reduction in *SOX10* and *MAGEA3* transcript levels, but no reduction in the levels of *AMIGO2* or any of the three reference genes used to normalise qRT-PCR data (*GAPDH*, *RPS13* and *TBP*). Interestingly, we also observed significant reductions in the transcript abundance of *LINC00511*, *LINC01234* and *TMEM51-AS1*, which represented the top three SFPQ-enriched lncRNA genes by cell type specificity, however, we did not detect any change in the expression of the melanoma-specific lncRNA, *SAMMSON*, which was not enriched with SFPQ in our RIP-seq dataset. Similarly, overexpression of SFPQ resulted in significant increases to *LINC00511* and *LINC01234* transcript levels, but no change in the levels of SAMMSON (Fig. S3). To assess if observed changes in SFPQ-enriched transcript levels following knockdown of SFPQ were related to post-transcriptional regulation, SFPQ was depleted and RNA Pol II activity inhibited via treatment with actinomycin D, prior to analysis of target gene transcript levels over a 6 h period. Only *SOX10* and *TMEM51-AS1* exhibited decreased stability in SFPQ-depleted cells (Fig. 5b). To gain a better understanding of how SFPQ might be regulating the expression of enriched RNAs, SFPQ-enriched transcript regions were identified and annotated, and Integrative Genomics Viewer utilised to visualise coverage of the reads across the identified genes. Global analysis of SFPQ peaks revealed a similar profile in PM and A2058 melanoma cells, with most binding occurring at exon–intron boundaries and towards the 3′end of transcripts (Fig. 5c and Fig. S4). Together, these data demonstrate that SFPQ contributes to the efficient expression of several melanoma-associated oncogenic transcripts in a predominantly RNA stability-independent manner, and by doing so contributes to the maintenance of a ‘cancer transcriptomic state’ in melanoma cells, in vitro.

### SFPQ is a potential prognostic biomarker for melanoma

**Fig. 6:**
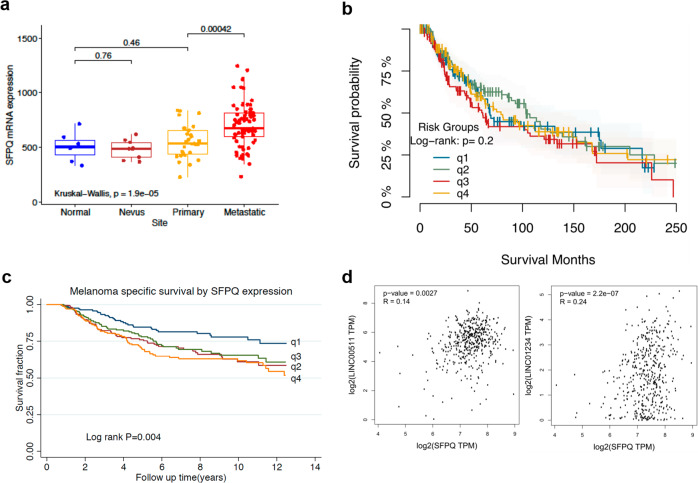
Increased expression of SFPQ in primary melanoma is associated with decreased melanoma patient survival. **a** GEO data (GSE46517) was analysed to determine SFPQ mRNA expression in normal skin, benign nevi, primary and metastatic melanoma. **b** SFPQ expression did not associate with patient survival in TCGA-melanoma datasets. **c** Patients with lower SFPQ expression in primary tumours have an improved melanoma-specific survival in the LMC. Data presented in quartiles, q1–q4. **d** Expression correlation was analysed for SFPQ, *LINC00511* and *LINC01234* using TCGA-melanoma datasets on the GEPIA2 web portal.

Analysis of data detailing SFPQ expression in normal skin compared with nevi, primary and metastatic melanoma (GSE46517) was carried out and revealed that SFPQ expression is significantly increased in metastatic tumours compared with control and primary tumour samples (Fig. 6a). However, SFPQ expression groups (stratified by expression quantiles) were not associated with patient survival in the TCGA-melanoma dataset (Fig. 6b). To investigate if SFPQ expression groups in primary melanoma tumours were prognostic for survival, we utilised our previously published transcriptomic data from the primary melanomas of 703 patients, which comprises part of the Leeds Melanoma Cohort (LMC) [36]. Analysis of this cohort revealed that primary tumour expression of SFPQ significantly predicted patient survival with reduced survival for patients in the top three quartiles compared with the lowest quartile (Fig. 6c). Unfortunately, the HT12.4 array utilised does not contain probes for any of the melanoma-specific SFQP-enriched lncRNA described in this study, preventing analysis of *LINC00511* and *LINC01234* expression and patient survival in primary melanoma. Although less relevant, two PM-specific SFPQ lncRNA interactors were available for analysis in the LMC cohort and interestingly their expression was positively associated with patient survival (Fig. S5). To determine if SFPQ expression correlated with expression of *LINC00511* and *LINC01234* in melanoma tumours, we analysed TCGA-melanoma datasets on the GEPIA2 web portal [37] and found significant correlation between the expression of SFPQ and *LINC00511* (*r* = 0.14, *p* = 0.0027), and SFPQ and *LINC01234* (*r* = 0.24, *p* = 2.2^−07^) (Fig. 6d), suggesting that these lncRNAs are positively associated with SFPQ. Together, these data demonstrate that increased expression of SFPQ in primary melanoma is associated with decreased melanoma patient survival and that SFPQ expression may have some utility as prognostic biomarker in primary melanoma.

## Discussion

The precise role of SFPQ in cancer is complex and appears to be tissue specific. For example, in breast and prostate cancer SFPQ appears to be an important driver of the cancer phenotype via post-transcriptional regulation of key genes such as oestrogen receptor α (ERα) and androgen receptor (AR), respectively [17, 20]. Whereas in colorectal, lung and renal cancer SFPQ has been reported to act as a transcriptional repressor that serves to dampen proto-oncogene expression, a function that is frequently derailed via the overexpression and binding of oncogenic lncRNAs, such as *MALAT-1* [38] and *SANT1* [15]. The role of SFPQ in melanoma is less clear, with a single article suggesting SFPQ attenuates *RAB23* expression via binding of the lncRNA, *LLME23* [39]. Interestingly, we did not detect the *LLME23* transcript in our A2058 RIP-seq dataset. Indeed, the *LLME23* transcript remains incompletely annotated and is unlisted as a lncRNA in both the NCBI 109.20200815 and Ensembl 101 genome releases, casting some doubt on the nature of this gene.

Data in Fig. 1b–f suggest that the net functional output of SFPQ expression in melanoma cell lines is oncogenic. This hypothesis is supported by studies in hormone-refractory prostate cancer, where increased SFPQ expression has been reported to drive expression of *AR* transcripts, in addition to other key driver genes, to promote disease progression (17). RIP-seq analysis demonstrated that the SFPQ-RNA interactomes of PM and melanoma cells diverged significantly and, crucially, in a manner that was not simply associated with relative transcript abundance (Fig. 2c), which can be a limitation of RIP-seq, suggesting that these differences were due to reprogramming. Interestingly, gene-level analysis of SFPQ-enriched mRNA transcripts exclusively associated with PM revealed several targets that are frequently downregulated in melanoma and specifically associated with ECM architecture (COL1A2, COL3A1, Decorin, and Fubilin-1) (40), in addition to the proapoptotic tumour suppressor gene, DPP4, which has been reported as a suppressor of metastatic progression in melanoma [40]. In contrast, the top SFPQ-enriched mRNA transcripts in melanoma cells were generally associated with disease progression, such as *AMIGO2*, *MAGEA3*, *MAGEA1*, and the transcription factor, *SOX10* [41–45].

A similar story emerged when we compared lncRNA enriched with SFPQ in PM and melanoma cell backgrounds. The SFPQ-enriched lncRNA transcripts most specific to PM were *EMX2OS* and *FENDRR*, the latter reported to function as a tumour suppressor in most cancers, including melanoma [27, 46–49]. The function of *EMX2OS* in cancer differs depending on cell background [28, 50]. We observed a downregulation of *EMX2OS* in several melanoma cell lines, compared with PM (Fig. S5). Moreover, analysis of LMC patient data demonstrated an inverse relationship between *EMX2OS* expression and tumour thickness, mitotic rate, and survival (Fig. S5), suggesting that this lncRNA may function as a tumour suppressor in melanoma. In contrast, A2058-specific SFPQ-enriched lncRNA included *LINC00511*, *DUXAP8*, *TMEM51-AS1*, *FOXD2-AS1* and *LINC01234*, all of which have been shown to drive metastasis in a range of cancers via various mechanisms [29, 30, 51–55], however, only *FOXD2-AS1* has been associated previously with melanoma [54].

Our analysis of two SFPQ-enriched lncRNAs, *LINC00511* and *LINC01234*, both with established roles as oncogenes in other cancer backgrounds, revealed that knockdown of these genes impaired melanoma viable cell growth, migration and, in the case of *LINC00511*, cellular respiration (Fig. 3b–f). Recently, *LINC00511* was reported to accumulate in both nuclear and cytoplasmic fractions [33], suggesting that this lncRNA may possess sponging activity. We demonstrated that *LINC00511* expression is inversely correlated with miR-625-5p transcript levels (Fig. 4b) and that disruption of the *LINC00511*/miR-625-5p axis resulted in decreased expression of *PKM2* (Fig. 4c–e), presumably via its well-defined role in regulating glycolytic flux [56]. *PKM2* was absent from the list of SFPQ-enriched transcripts in our RIP-seq data and SFPQ knockdown had no impact on *PKM* expression or exon 9–10 splicing (Fig. 4c and Fig. S2), suggesting that it does not regulate alternative splicing of *PKM*. This result was somewhat unexpected, as SFPQ knockdown does reduce the expression of *LINC00511* (Fig. 5a). This observation may relate to partial knockdown of SFPQ protein (~50%) via ASO-transfection, which resulted in an ~40% decrease in *LINC00511* transcript levels (Fig. 5a) compared with the ~97% knockdown achieved by directly targeting *LINC00511* with ASOs (Fig. 4b). Therefore, it seems possible that while SFPQ knockdown leads to a significant decrease in the expression of *LINC00511*, this is not sufficient to dysregulate the *LINC00511*/miR-625-5p axis and thus does not impact on *PKM2* expression.

Our observation that melanoma-specific SFPQ-enriched genes were significantly decreased following SFPQ knockdown (Fig. 5a) and increased in cells overexpressing FLAG-SFPQ (Fig. S3) supports a role for SFPQ in efficient expression of bound-transcripts, rather than endogenous post-transcriptional processing events. Indeed, a recent article demonstrating that diminished availability of splicing factors in the nucleoplasm attenuates transcript release from the parent gene [57] provides a model whereby SFPQ may positively contribute to target RNA expression, a hypothesis that is particularly appealing given that SFPQ is a key regulator of transcriptional termination [58]. A second model, that is not mutually exclusive, is that SFPQ may regulate the expression of bound RNAs via increased stabilisation of the transcript. SFPQ has been previously shown to regulate the stability of *AR* transcripts in prostate cancer and neurons and our own analysis of SFPQ-enriched transcripts identify *SOX10* and the lncRNA, *TMEM51-AS1* as being destabilised in SFPQ depleted cells (Fig. 5b). How SFPQ mediates transcript stability remains unclear, but one possibility is that SFPQ shields bound RNAs from degradation, in a manner analogous to other RNA-binding proteins (RBP), such as Human antigen R (HuR) [59, 60] or the La-related protein superfamily [61, 62].

Analysis of a large population-based melanoma cohort demonstrated that melanoma patients with increased expression of SFPQ in primary tumours were significantly more likely to die due to their disease (Fig. 6c). However, while SFPQ expression was significantly increased in metastatic melanoma, compared with primary tumours (Fig. 6a), analysis of TCGA-melanoma datasets showed that differential expression of SFPQ in metastatic disease was not associated with patient survival (Fig. 6b). There are several differences between the LMC and TCGA cohorts. The most obvious is that LMC tumours are all primaries at different AJCC stages (~35% stage I, 50% stage II and 15% stage III) while TCGA’s are mostly metastases (>80%). Moreover, LMC samples are population based (patients were recruited as they came into clinic), while TCGA samples are selected. Finally, in the LMC cohort cause of death and subsequently analysed survival was exclusive to melanoma-associated deaths. Thus, the different results observed in the LMC and TCGA datasets suggest that high expression of SFPQ in primary tumours might be a driver of disease progression and thus have some utility as a prognostic biomarker, but at late-stage disease, further increases in SFPQ expression are less significant.

In summary, we have shown that SFPQ positively contributes to the cancer phenotype in melanoma, likely via reprograming of the SFPQ-RNA interactome in melanoma to favour the expression of oncogenic transcripts. It will be interesting to determine the global impact of SFPQ knockout on RNA expression in melanoma, particularly given recent work in renal cancer demonstrating that RBPs have a wide-ranging impact on RNA expression programs and by extension the cancer transcriptome [63].

## Materials and methods

### Tissue culture

Immortalised cell lines were obtained from European Collection of Authenticated Cell Cultures and certified mycoplasma-free. UACC-62 and A375 cells were maintained in Minimum Essential Medium, M14 in Roswell Park Memorial Institute 1640 Medium (RPMI), and A2058 cells in Dulbecco’s Modified Eagle Medium, in each case supplemented with 10% foetal bovine serum, 100 U/ml of penicillin and 100 µg/ml of streptomycin. Primary human melanocytes (CELLnTEC, Switzerland) were grown in a 2:1 mixture of keratinocyte serum-free media (K-SFM) and Eagle’s minimal essential medium supplemented with 25 µg/ml bovine pituitary extract, 0.2 ng/ml rEGF, penicillin (100 U/ml), streptomycin (100 µg/ml) and 2 mM l-glutamine. All cells were cultured at 37 °C and 5% CO_2._ LNA inhibitors were transfected into cells using HiPerfect (QIAgen, UK), as previously described [64]. Details of LNA inhibitors used in this study can be found in Table S1, pSFPQ-FLAG has been previously described [65].

### Cell migration

For wound healing assays scratches were generated in a confluent cell sheet using a 200 μl pipette tip and cells cultured in SFM. Images of each wound were captured over 24 h (EVOS XL Core Cell Imaging System, 4× objective) and wound closure measured using the MRI tool (Image J). Transwell migration assays were carried out in 8 µm inserts (Starstedt, Germany) and migrated cells stained using 0.2% (w/v) crystal violet (Fisher Science, UK), imaged (EVOS XL Core Cell Imaging System) and the number of migrated cells calculated, as previously described [66].

### Viable cell growth

Cells were seeded at a density of 1 × 10^4^ cells/well in white-walled 96-well plates (ThermoFisher, UK). After 24 h, media was replaced with 100 μl of SFM and cells incubated at 37 °C for a further 24, 48 and 72 h with viable cell growth assessed at each time point using CellTiterGlo Luminescent Cell Viability Assay (Promega, UK) and luminescence recorded 10 min after reagent addition using the GloMax® Explorer system (Promega, UK).

### Apoptosis

Apoptosis and cell death was analysed via FACS using the Guava® easyCyte™ Flow Cytometer (Luminex, US), as described elsewhere [67]. For analysis of caspase 3/7 activity, cells were seeded at a density of 1 × 10^4^ cells/well, cultured for 24 h prior to addition of 100 μl of Caspase-Glo® 3/7 reagent directly to cells, incubation for 1 h at RT and recording of luminescence using the GloMax® Explorer system (Promega, UK).

### Analysis of cellular respiration

OCR was determined using the Mito Stress Test Kit and XFe96 Extracellular Flux Analyzer (Seahorse Bioscience, Billerica, MA, USA). Cells were seeded in Seahorse XF96 V3 PS Cell Culture Microplates (Agilent Technologies, UK). Following incubation, cells were transferred to unbuffered XF assay media at pH 7.4 supplemented with 25 mM glucose and 1 mM sodium pyruvate and placed at 37 °C in a non-CO_2_ incubator for one hour prior to assay. Cells were analysed using the Seahorse XF Cell Mito Stress Test (Agilent Technologies, UK). Immediately following each run cells were lysed, and protein concentration determined by Sulforhodamine B assay for normalisation. OCR was automatically calculated and analysed by the Seahorse Wave software (Agilent Technologies, UK).

### qRT-PCR

Total RNA was extracted from cells and 500 ng used to generate cDNA prior to qRT-PCR analysis using SsoAdvanced SYBR master mix (Bio-Rad, UK) on the CFX96 system (Bio-Rad, UK) normalised against *TBP*, *RPS13* and *GAPDH* reference genes, as described elsewhere (66). RNA stability assays were carried out using 1 μg/ml actinomycin-D, as previously described [68]. Micro-RNA expression was analysed using miRCURY LNA miRNA PCR assays (Qiagen, UK) and data normalised against *SNORD44* and *SNORD48* reference genes. A list of oligonucleotides used during this study can be found in Table S2.

### RIP-seq

SFPQ-RNA immunoprecipitations were performed using SFPQ-specific antibody or IgG control (Table S3) and the Magna RIP™ RNA-Binding Protein Immunoprecipitation Kit (Merck Millipore, USA). Total RNA libraries were prepared using TruSeq Stranded Total RNA Sample Prep Kit (Illumina, USA) and the TruSeq cDNA libraries were analysed via NextSeq 500 (Illumina, USA) and data deposited at GEA (GSE171291). Details of RIP-seq data analysis are available in Supplementary Methods.

### Cell dependency analysis

Gene dependency data for 18120 across 54 Melanoma cell lines were download from DepMap project (https://depmap.org/portal/). DEMETER2 inferred z-scores representing cell viability independent gene knockdown effects and expression values were extracted for SFPQ.

### Clinical data

The LMC transcriptomic data comprises 703 primaries profiled on Illumina DASL array HT12.4 (EGAS00001002922, [36, 69]). Correlation between tumour characteristics and tumour expression of targets was performed via Spearman rank correlation. Data from TCGA-SKCM [70] were analysed to determine the correlation between gene expression, copy number alteration and patient clinical characteristics. These expressions were further tested for association with melanoma-specific survival after their split into quartiles by applying Cox proportional hazards regression and plotting Kaplan-Meier survival curves. Where appropriate, certain quartiles were combined to reduce the number of parameters. Both single gene models and bivariate models were applied with an interaction term to test the independence between genes. These analyses were conducted in STATA v14 (StataCorp, TX, USA), or R using tools provided on the GEPIA2 web portal http://gepia2.cancer-pku.cn/#survival.

### Statistical analysis

Except otherwise stated, graphical data shown represent mean ± standard error of mean (SEM) using at least three independent experiments. Differences between means was analysed by Student’s *t* test, Mann–Whitney Wilcoxon test, Kruskal–Wallis test, or one-way ANOVA as detailed in the figure legends. Survival analyses were conducted using the Cox proportional hazards regression and Kaplan–Meier graphs with log-rank test. Statistics was considered significant at *p* < 0.05.

## Supplementary information

Table S1

Table S2

Table S3

Fig S1

Fig S2

Fig S3

Fig S4

Fig S5

Supplementary Methods
