# Supplementary material for: SFPQ promotes an oncogenic transcriptomic state in melanoma
Source: Oncogene. 2021 Jul 3;40(33):5192–203. doi: 10.1038/s41388-021-01912-4 (PMC8376646; doi:10.1038/s41388-021-01912-4)
Supplement: Supplementary file 1 — Table S1 [file 41388_2021_1912_MOESM1_ESM.docx]

**Table S1**

| Target | Sequence |
| --- | --- |
| Negative Control | AACACGTCTATACGC |
| *SFPQ*#1 | AGCTTAATAAATCCGA |
| *SFPQ*#2 | TCGTCCAGTCATTGTGG |
| *LINC00511*#1 | TTCTAACCTGCATCAT |
| *LINC00511*#2 | CGGAGTGTGGTAAGAA |
| *LINC01234*#1 | ACGGAGCTTTTTAAGT |
| *LINC01234*#2 | ATGTCTCCTCTGCGTG |
