# Supplementary material for: SFPQ promotes an oncogenic transcriptomic state in melanoma
Source: Oncogene. 2021 Jul 3;40(33):5192–203. doi: 10.1038/s41388-021-01912-4 (PMC8376646; doi:10.1038/s41388-021-01912-4)
Supplement: Supplementary file 2 — Table S2 [file 41388_2021_1912_MOESM2_ESM.docx]

| Antibody | Supplier | Catalogue number |
| --- | --- | --- |
| SFPQ | TGGACAACAGAGCGAGAC | AACAGAAGTAGCACAAGGAGAT |
| SOX10 | CAGTGGTATTTGAATAAAGTATG | CAGGAGACAGTAATGAGTT |
| AMIGO2 | TTCTGGATTCTGAGTGGATTC | TGCTGGTGATGTTGTTATGA |
| MAGEA3 | GCAGTCAGCATTCTTAGT | CTCATTCAACCATCCGTTA |
| LINC00511 | TATAATGCCTAACACAAC | TGGATATAAATATACATAGTCA |
| TMEM51-AS1 | ATATCGTTAGTCTGATTATTCCT | CACACTTCCTCAACTCTC |
| LINC01234 | TCCCACAAAACAACCACCCA | GTACCCTGTGAGTCAGTGGC |
| SAMMSON | TTCCTCAACTATGCAACTCAA | TAGACTACGGGCTCATGACTT |
| PKM1 | TCACTCCACAGACCTCATGG | GAAGATGCCACGGTACAGGT |
| PKM2 | ATCGTCCTCACCAAGTCTGG | GAAGATGCCACGGTACAGGT |
| TBP | CATGCTTGACACTTGGTGCC | GGTCGCAGGTGGATCTCTTC |
| GAPDH | TTTGACACGAGTGACTGTATTTTGAA | ATTTATGAATTGTCACAGGACCTCT |
| RPS13 | GGTTGAAGTTGACATCTG | ATCTGTGAAGGAGTAAGG |
| miR-625-5p | AGGGGGAAAGUUCUAUAGUCC |  |

**Table S2**
