# Supplementary material for: SFPQ promotes an oncogenic transcriptomic state in melanoma
Source: Oncogene. 2021 Jul 3;40(33):5192–203. doi: 10.1038/s41388-021-01912-4 (PMC8376646; doi:10.1038/s41388-021-01912-4)
Supplement: Supplementary file 3 — Table S3 [file 41388_2021_1912_MOESM3_ESM.docx]

| Antibody | Supplier | Catalogue number | Dilution |
| --- | --- | --- | --- |
| SFPQ | Abcam | Ab38148 | 1:1000 |
| Rabbit IgG | Abcam | Ab171870 | (ChIP-grade used in RIP) |
| GAPDH | Cell Signalling Technology | 97166 | 1:10000 |
| E-cadherin | Cell Signalling Technology | 3195T | 1:1000 |
| N-Cadherin | Cell Signalling Technology | 13116T | 1:1000 |
| FLAG | Merck (Sigma-Aldrich) | F3165 | 1:10000 |
| Vimentin | Cell Signalling Technology | 5741S | 1:1000 |

**Table S3**
