# Supplementary material for: SFPQ promotes an oncogenic transcriptomic state in melanoma
Source: Oncogene. 2021 Jul 3;40(33):5192–203. doi: 10.1038/s41388-021-01912-4 (PMC8376646; doi:10.1038/s41388-021-01912-4)
Supplement: Supplementary file 4 — Fig S1 [file 41388_2021_1912_MOESM4_ESM.pdf]

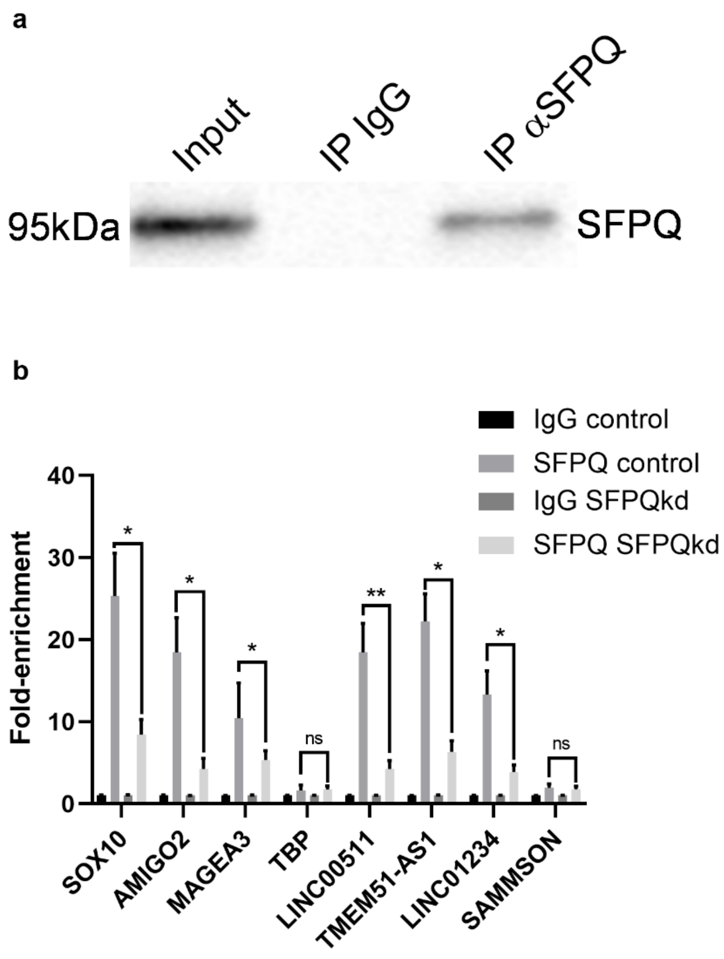

Fig. S1

a Protein was isolated from cell lysates (Input) and magnetic beads (IP) prior to and after RNA-IP, respectively and SFPQ detected via immunoblotting with SFPQ-specific antibodies. Input = 5% of total lysate used in RNA-IP and IP samples represent 5% of magnetic bead slurry following IP and washes. **b** ASO-mediated knockdown of SFPQ in A2058 cells resulted in a reduction of transcript enrichment verses input when compared with control, indicating that enrichment of target genes was SFPQ-specific. A2058 cells were transfected with SFPQ-specific GapmeRs or control and cultured for 48h prior to RNA-IP using either an SFPQ-specific antibody or IgG-antibody isotype control. The relative enrichment of SFPQ-interacting transcripts compared to input was determined via qRT-PCR for both control and SFPQ-depleted cells, n=3.
