# Supplementary material for: SFPQ promotes an oncogenic transcriptomic state in melanoma
Source: Oncogene. 2021 Jul 3;40(33):5192–203. doi: 10.1038/s41388-021-01912-4 (PMC8376646; doi:10.1038/s41388-021-01912-4)
Supplement: Supplementary file 6 — Fig S3 [file 41388_2021_1912_MOESM6_ESM.pdf]

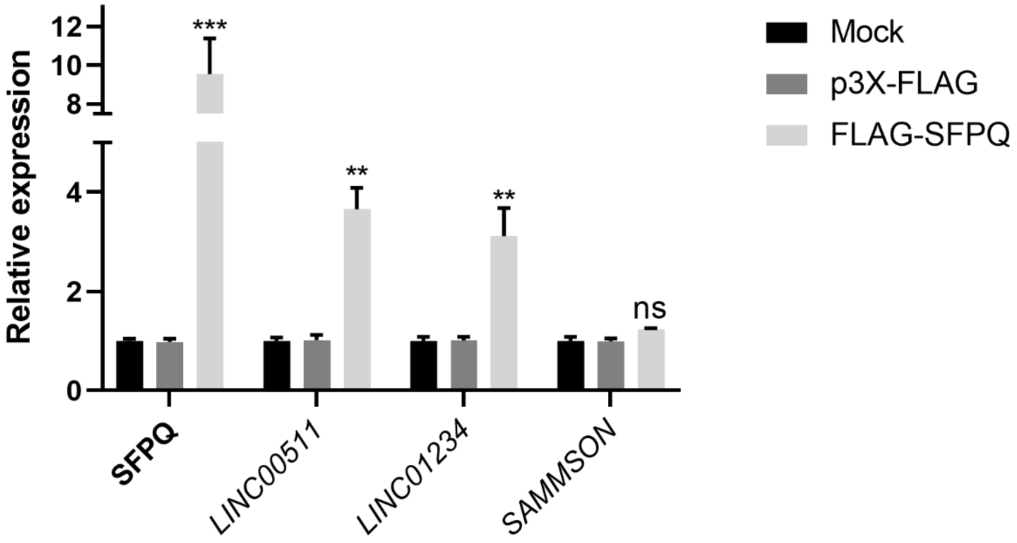

Fig. S3

A2058 cells were transiently transfected with pFLAG-SFPQ or parent vector control and cultured for 48h prior to isolation of total RNA and analysis of SFPQ, *LINC00511*, *LINC01234* and *SAMMSON* transcript levels via qRT-PCR, n=3.
