# Supplementary material for: SFPQ promotes an oncogenic transcriptomic state in melanoma
Source: Oncogene. 2021 Jul 3;40(33):5192–203. doi: 10.1038/s41388-021-01912-4 (PMC8376646; doi:10.1038/s41388-021-01912-4)
Supplement: Supplementary file 7 — Fig S4 [file 41388_2021_1912_MOESM7_ESM.pdf]

AMIGO2

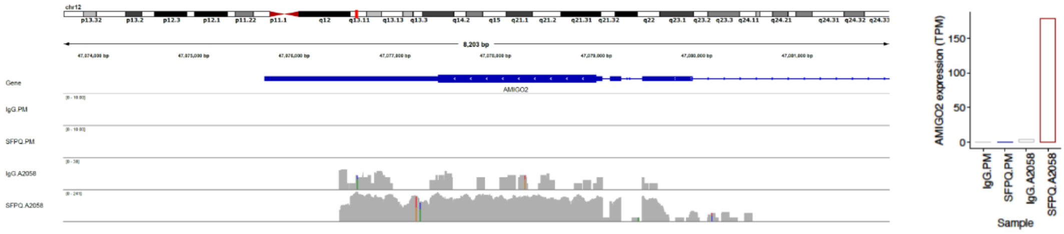

SOX10

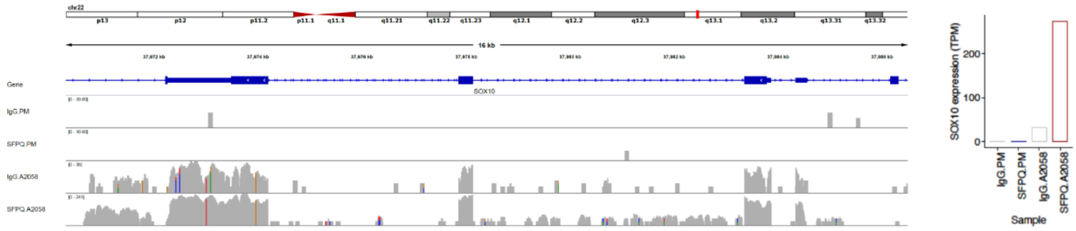

MAGEA3

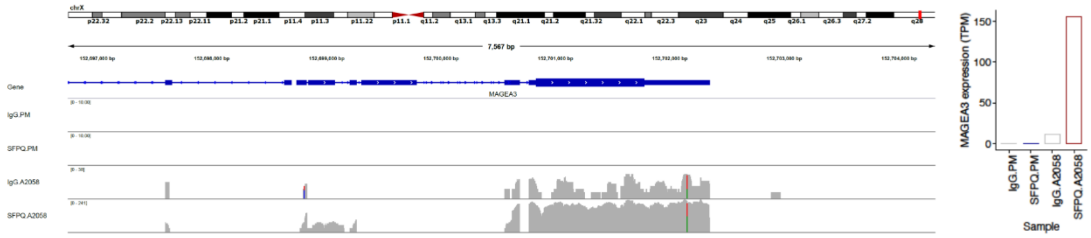

LINC00511

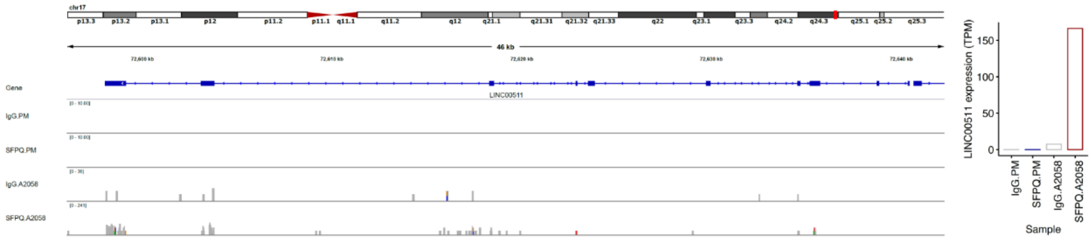

LINC01234

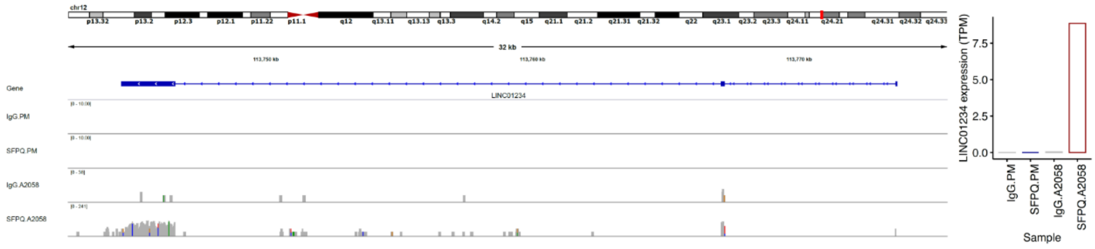

TMEM51-AS1

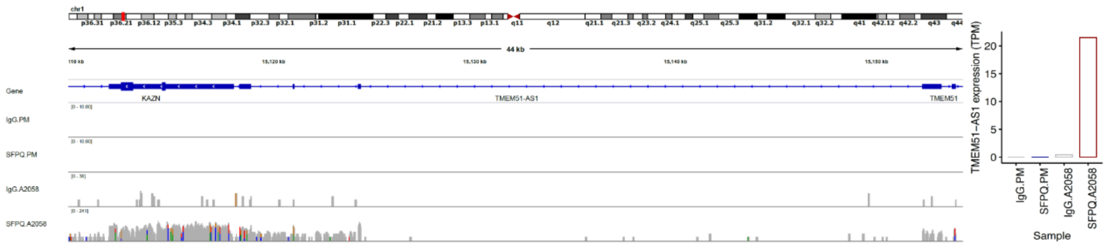

Fig. S4

Gene-level analysis of SFPQ identifies enhanced binding of RNA situated towards the 3'-end of enriched transcripts. SFPQ-IP peaks were annotated using the ChIPseeker R Bioconductor package and the Integrative Genomics Viewer (IGV) used to visualise the coverage of the reads across the identified genes.
