# Supplementary material for: SFPQ promotes an oncogenic transcriptomic state in melanoma
Source: Oncogene. 2021 Jul 3;40(33):5192–203. doi: 10.1038/s41388-021-01912-4 (PMC8376646; doi:10.1038/s41388-021-01912-4)
Supplement: Supplementary file 8 — Fig S5 [file 41388_2021_1912_MOESM8_ESM.pdf]

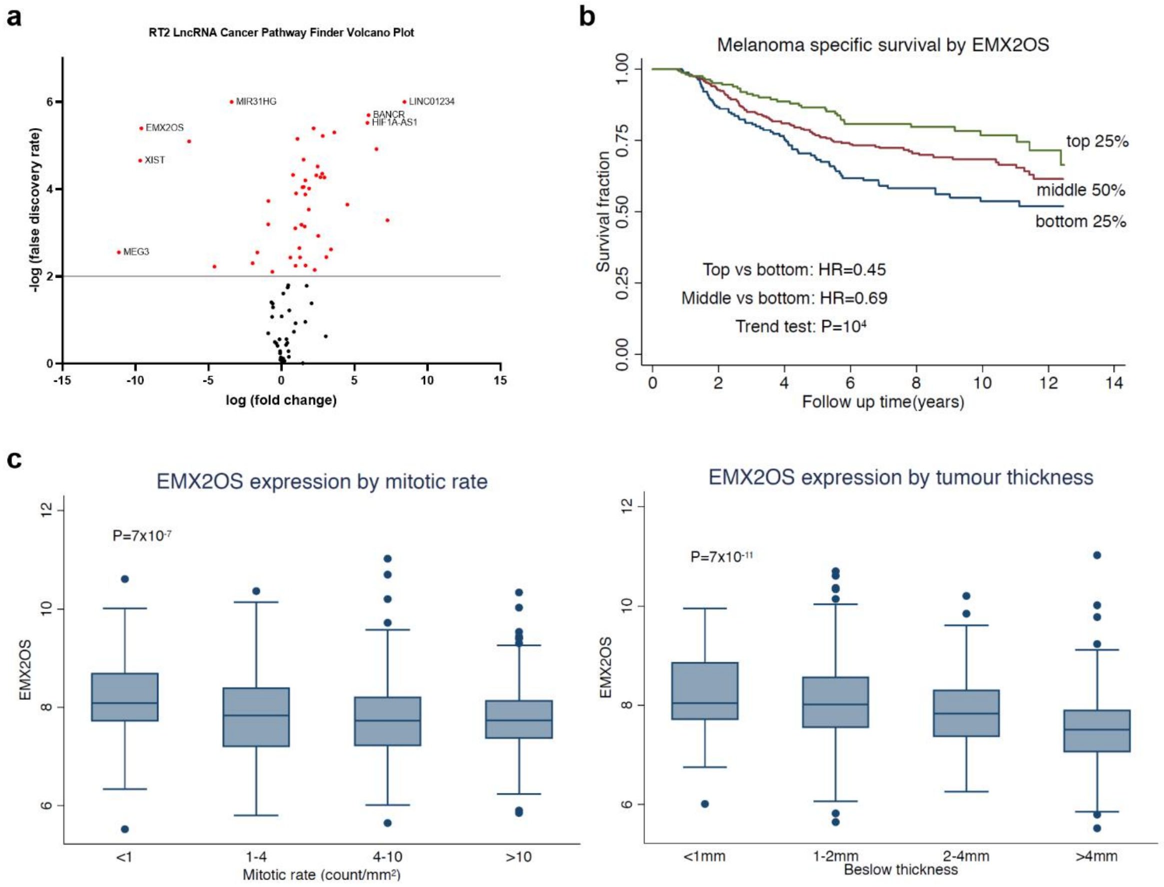

Fig. S5

a RT^2 IncRNA PCR Array Human Cancer Pathway Finder (Qiagen, UK) was used to determine the expression levels of lncRNA in A2058 cells compared with PM. The volcano plot displays log fold change and -log FDR for each gene. **b** Patients with higher expression of *EMX2OS* in the primary tumour have a prolonged melanoma-specific survival in the LMC. **c** Primary tumour expression of *EMXOS* is the strongest in thinner and less mitotic tumours (Kruskall-Wallis test).
