# Supplementary material for: SFPQ promotes an oncogenic transcriptomic state in melanoma
Source: Oncogene. 2021 Jul 3;40(33):5192–203. doi: 10.1038/s41388-021-01912-4 (PMC8376646; doi:10.1038/s41388-021-01912-4)
Supplement: Supplementary file 9 — Supplementary Methods [file 41388_2021_1912_MOESM9_ESM.docx]

**Supplementary Methods**

**RIP-seq**

Raw reads were subjected to adaptor trimming (Illumina single end sequencing adapters) and low-quality reads removal using Trimmomatic v0.39 [1] with parameters (windowSize = 4 and requiredQuality >20). Quality filtered, and adapter trimmed reads were aligned to the GRCh38/hg38 assembly of the human genome using HISAT2 v2.1.0 [2] with default parameters. StringTie v1.3.4 (default parameters) was used to assemble and quantify the RNA abundance based on the GRCh38 annotation (GENCODE Release 32). Expression levels were normalised by “transcripts per kilobase million” (TPM). Peaks were called against IgG control libraries at p<10^-5^ using MACS2 under the option of *-no model* with -*effective genome size* and *-shift size* set to 3.0 x 10^8^ and length of RNA fragments (150bp), respectively. To reduce the rate of false-positive enrichments, only uniquely mapped and non-duplicated reads were used for peak calling. SFPQ-IP peaks were annotated using the ChIPseeker R Bioconductor package [3] and the Integrative Genomics Viewer (IGV) used to visualise the coverage of the reads across the identified genes [4]. Biological processes associated with the differentially enriched genes were determined using R ClusterProfiler and the human Bioconductor annotation database (org.Hs.eg.db) to compare the enriched biological processes between PM-enriched, A2058-enriched, and shared genes. All enrichment analyses were performed with a strict p-value and q-value<0.01 cut-off with reduced redundancies by semantic similarity analysis [5].

1 Bolger AM, Lohse M, Usadel B. Trimmomatic: a flexible trimmer for Illumina sequence data. *Bioinformatics* 2014; 30: 2114-2120.

2 Kim D, Paggi JM, Park C, Bennett C, Salzberg SL. Graph-based genome alignment and genotyping with HISAT2 and HISAT-genotype. *Nat Biotechnol* 2019; 37: 907-915.

3 Yu G, Wang LG, He QY. ChIPseeker: an R/Bioconductor package for ChIP peak annotation, comparison and visualization. *Bioinformatics* 2015; 31: 2382-2383.

4 Thorvaldsdottir H, Robinson JT, Mesirov JP. Integrative Genomics Viewer (IGV): high-performance genomics data visualization and exploration. *Brief Bioinform* 2013; 14: 178-192.

5 Wang JZ, Du Z, Payattakool R, Yu PS, Chen CF. A new method to measure the semantic similarity of GO terms. *Bioinformatics* 2007; 23: 1274-1281.
